# Dynamic in Species Estimates of Carnivores (Leopard Cat, Red Fox, and North Chinese Leopard): A Multi-Year Assessment of Occupancy and Coexistence in the Tieqiaoshan Nature Reserve, Shanxi Province, China

**DOI:** 10.3390/ani10081333

**Published:** 2020-08-01

**Authors:** Kasereka Vitekere, Jiao Wang, Henry Karanja, Kahindo Tulizo Consolée, Guangshun Jiang, Yan Hua

**Affiliations:** 1 Feline Research Center of National Forestry and Grassland Administration, College of Wildlife and Natural Protected Area, Northeast Forestry University, Harbin 150040, China; vitekere@nefu.edu.cn (K.V.); wj15765526221@163.com (J.W.); hkaranja@egerton.ac.ke (H.K.); tulizok@yahoo.com (K.T.C.); 2 Department of Natural Resources, Egerton University, P.O. Box 536-20115 Egerton, Kenya; 3 Tayna Center for Conservation Biology, University of Nature Conservation and Development at Kasugho, Goma 167, North-Kivu, Democratic Republic of Congo; 4 Guangdong Provincial Key Laboratory of Silviculture, Protection and Utilization, Guangdong Academy of Forestry, Guangzhou 510520, China

**Keywords:** anthropogenic disturbances, colonization, environmental factor, extirpation, occupancy, species dynamics

## Abstract

**Simple Summary:**

Carnivores are among the threatened mammal species due to interactions with humans and environmental effects. We collected data using camera traps across three sampling periods over three years, aiming to study the coexistence mechanisms of three carnivores: the North Chinese leopard, the leopard cat, and the red fox to depict effects from an environmental factor and anthropogenic disturbances on these carnivores within a landscape. The occupancy modeling showed that all species’ occupancy did not greatly change across years and they rather depicted occupancy stability. The elevation impacted more on species’ occupancy estimates than distances to villages and roads. We performed a multi-year assessment of species’ estimates and the results of this study reveal the impact of habitat features and anthropogenic disturbances on the occupancy dynamics of the North Chinese leopard, the red fox and the leopard cat in the Tieqiaoshan nature reserve landscape.

**Abstract:**

Wildlife populations are spatially controlled and undergo frequent fluctuations in abundance and site occupation. A comprehensive understanding of dynamic species processes is essential for making appropriate wildlife management plans. Here, we used a multi-season model to describe the dynamics of occupancy estimates of the carnivores: North Chinese leopard (*Panthera pardus japonensis*, Gray, 1862), leopard cat (*Prionailurus bengalensis*, Kerr, 1792), and red fox (*Vulpes vulpes*, Linnaeus, 1758) in the Tieqiaoshan Nature Reserve, Shanxi Province, China, over a three-year study period using camera traps data. The occupancy probability of the North Chinese leopard did not markedly change with time as the occupancy equilibrium was constant or slightly enhanced. The occupancy of the leopard cat decreased with time. The occupancy equilibrium of the red fox alternately increased and decreased. However, all species presented a slight level of occupancy stability due to their small values of the rate of change in occupancy. Environmental factor and anthropogenic disturbances slightly influenced the occupancy of all species and the colonization and extirpation probability of the red fox. The colonization and extirpation for all species were relatively more strongly affected by the distances to villages and roads. Moreover, elevation increased the colonization and decreased the extirpation for the leopard cat. Species interaction factors increased with time for all species. The North Chinese leopard and the leopard cat avoided each other. The leopard cat and the red fox independently co-occurred. There was true coexistence between the North Chinese leopard and the red fox. This research confirmed that environmental factors and human perturbations are vital factors to consider in wild carnivores’ conservation and management.

## 1. Introduction

In natural habitats, the abundance of a population within a habitat may fluctuate [1]. Spatiotemporal patterns are characteristic of species dynamics. Demographic parameters such as birth and death rates and migrations modulate population distributions by alternately increasing and decreasing the number of individuals [2,3]. Therefore, the use of the site by the population varies with time. Indeed, spatial patterns are also crucial as habitat loss by modification and fragmentation disrupts local cohesion and alters individual occupancy [4,5].

Within landscapes, species may be relatively numerous at some sites and in certain habitats (forest groves or close to the salt marshes). At other locations, however, they may be scarce (disturbed habitats in vicinity with human activities) compared to their core distribution [1]. Wildlife managers must prioritize habitat integrity in order to maintain the species in protected areas [6,7,8] and ensure longtime wildlife presence in protected areas [9]. Effective wildlife conservation requires understanding the mechanisms that cause population fluctuations. Site occupation is measured to determine how species react to habitat changes caused by environmental or anthropogenic perturbations [10]. Therefore, in wildlife management, it is essential to assess the impacts of environmental factors and anthropogenic disturbance variations in a landscape on the viability of species.

Most large mammals are particularly vulnerable to habitat change as they have naturally low birth rate, are more hunted than other species, and perform large-range movements for resources [11]. Landscape changes threaten mammal occupancy and big predators such as *Pathera* spp. require wide living spaces for their daily activity. In the attempt to meet their resource requirements, carnivores may extend their activity beyond protected areas [3,12] where they might encounter human land use [13]. In China, anthropogenic threats have degraded 63–75% of leopard (*Panthera pardus*) habitat [14,15].

The ecology of carnivore species has been extensively researched. These studies showed that carnivores have a substantial impact on animal population structure in a habitat [3,16], trophic level function [17], ecosystem resilience [18], and prey population stability [19]. Thus, ecologic studies of carnivores are vital within an ecosystem, and carnivores site occupation is an important factor in these studies, particularly for species distribution. The correlation between changes in local population and the species distribution narrows the description of spatiotemporal population dynamics. Nevertheless, elucidation of these processes is important for effective species management [20,21]. Changes in habitat occupancy over time reliably indicate extirpation or colonization. These changes can be evaluated by statistical models [22].

In most habitats, sites where natural vegetation predominates are the most preferred by carnivore populations [3]. At such locations, most native species still occur and ecological processes (predation, niche partitioning, etc.) operate essentially unchanged [3]. Our study area, the Tieqiaoshan Natural Reserve (TNR), is a habitat for sympatric carnivores such as the endangered North Chinese leopard (NCL), which is endemic to China [5,23,24], a large carnivore with approximatively 32–80 kg. The NCL should occupy specific ranges with high vegetation cover rate [25]. The areas surrounding the TNR are adversely affected by human disturbances (land transformation, fragmentation of natural ecosystems, implanting built up areas, infrastructure, husbandry, etc.). Consequently, carnivore population sources in the reserve’s favorable habitat may be transformed into population sinks and destabilize carnivore distributions within the reserve. The NCL regularly interacts with the red fox (RF) (*Vulpes vulpes*, Linnaeus, 1758) and the leopard cat (LC) (*Prionailurus bengalensis*, Kerr, 1792), but their ecological coexistence in this landscape remains unknown. The RF is the largest of the true foxes (2.2–14 kg), a Least Concern species, and one of the most broadly distributed carnivorous species. RF is distributed over the entire northern hemisphere [26], occupies various habitats, and is well adapted to human environments. The LC is a small wild cat (2–4 kg) native to East and Southeast Asia [27]. It is also a Least Concern species [28]. LC dwells in a wide range of natural habitats and modified areas [29]. There is a lack of information about population numbers of all three species in this area. We assumed that as NCL is a large carnivore, it should dominate smaller carnivores such as LC and RF. The information derived therefrom could help develop conservation measures for these three species in the study area. These three species were chosen in the TNR because they are the biggest carnivore species caught in our cameras within the TNR landscape, thus they would have an essential role in the ecological processes in the landscape, and have always been encountered in vicinity of human settlements and roads, according to our observations. We assumed that RF would dominate the LC. Environmental (elevation, habitat type, etc.) [12] and anthropogenic (human settlements and roads) [30,31] factors influence the spatiotemporal dynamics of a species. Dynamic estimates for these carnivores would be strongly correlated to environmental factors and anthropogenic disturbances. The influence of the co-occurrence of NCL, RF, and LC may be apparent in the estimates for all three species over time. Overlapping activity may enable a subordinate species to decrease the fitness of a dominant species [32,33] and increase the extirpation probability [34,35].

Several earlier multi-year assessments of species dynamics have focused mainly on occupancy, detection, colonization, and extirpation [16,36,37]. However, they did not attempt to clarify the mechanisms driving the rate of changes in site occupation of a species [2,38]. Some studies have used camera trap data to estimate species dynamics by occupancy over time [39]. Here, we tested the a priori hypothesis that both variable categories affect species estimates at different intensities over time. To this end, we applied multi-season occupancy models with covariates to disclose how an anthropogenic or environmental factor influences NCL, RF, and LC in their habitats.

We examined whether the recent conservation approaches applied to protect the TNR effectively mitigated changes in NCL, RF, and LC occupancy by reducing pressures across three years. We hypothesized that the rate of change in occupancy fluctuate and occupancy equilibrium will not be established for all three species. We also predicted that NCL, RF, and LC would be more profoundly influenced by anthropogenic disturbances than environmental factor during the same data collection period. Finally, we hypothesized that NCL, RF, and LC truly co-occurred and spatially overlapped.

## 2. Materials and Methods 

### 2.1. Study Area

**Figure 1:**
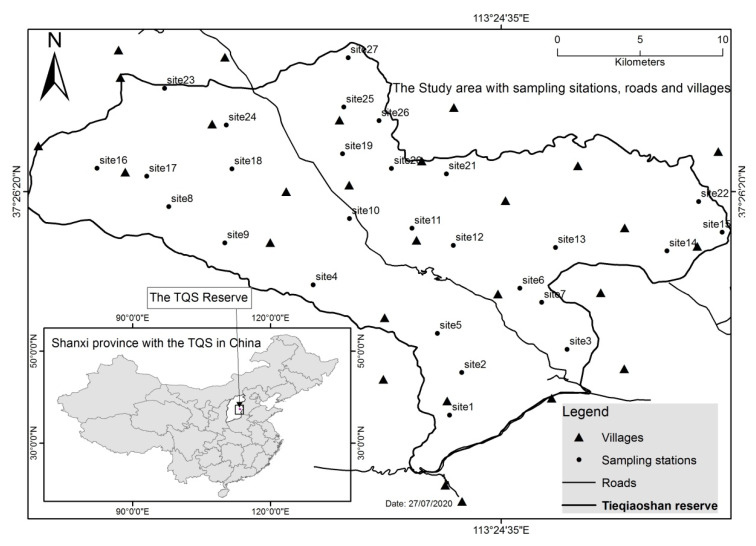
Overview of the study area. Black dots represent sites where camera traps were placed.

The present study was conducted at the Tieqiaoshan Nature Reserve (TNR), Shanxi Province, China (Figure 1). The TNR is a federally protected area created in 2002 by the federal people’s administration, allowing local population settlement in the TNR with strict measures of wildlife protection. The TNR has more than 40 villages, with nearly 30,022 inhabitants; the density is 85 inhabitants/km^2^. The total area is 353.52 km^2^; the elevation is mainly in the range of 1300–1700 m.a.s.l., with the highest point of 1827 m.a.s.l. The area hosts a high wildlife diversity including 24 species of mammals, 6 species of reptiles, 3 species of amphibians, and 116 species of birds. Sixteen wild animal species are protected there [23]. The region has a typical temperate continental monsoon climate with long winters and hot summers. The annual average temperature is almost 6 °C and the daily average is 10 °C [40]. The mean annual precipitation is 560 mm. Rainfall is intense from July to September. The soil is constituted by a mixture of sandy and stony parts. The vegetation is dominated by deciduous broadleaf, coniferous, and mixed deciduous forests. The dominant tree species are Chinese red pine (*Pinus tabuliformis*), Liaotung oak (*Quercus liaotungensis*), white birch (*Betula platyphylla*), and sparse North China larch (*Larix principis*). The TNR landscape has 78 genera of wild seed plants [40] and some grassland [25]. Within the TNR landscape, the wild boar (*Sus scrofa*), Eastern roe deer (*Capreolus pygargus*), and Cape hare (*Lepus sp*) are potential preys of the studied carnivores, mostly the NCL [23], as the RF and LC always prey on small mammals (Reserve Manager, Unpubl. data). Only an overall number of mammals is cited but specific statistics on NCL’s main prey remain unknown.

### 2.2. Study Design

#### 2.2.1. Presence/Absence Data

This investigation was a trap-based camera study. Presence and absence data were collected along with a variety of ancillary information (e.g., behavior, age, and physical characteristics). Cameras were deployed to take species’ photographs in a manner that is consistent with the sampling framework to yield data that are well suited for occupancy models [39]. Two brands of cameras were used, namely Eastern Red Hawk E1B 6210M and LTL6210MM, with only the first brand in the first sampling period and both brands in two other sampling periods. Data were gathered for three years (2017–2019) and corresponded to three sampling periods (seasons) with 130 (March–July 2017), 119 (September–December 2018), and 134 (March–June 2019) consecutive days, respectively. Recall that in the occupancy model the word “season” does not necessarily mean geographic season (winter, summer, autumn, or spring). It just means the period in which data were collected [34]. Eighty-one cameras were used in the first sampling period and 62 were used in the second and third sampling periods. Cameras were installed in an approximately 4 km × 4 km grid and attached to trees with average height = 0.50 m. Two or three cameras were deployed at each sampling site and faced each other on the animal trails [41]. The analyses were at the scale of camera trap stations rather than home ranges, as carnivore species were likely to range between different stations during the survey period. Cameras were set to capture date and time automatically; they were operated 24 h/day and the batteries were checked monthly. To ensure balanced sample representation at every site, each camera operated for ≥100 consecutive days per sampling period. A species was considered to be present on a sampling day if it appeared at least once in a single camera at a sampling site. To maintain temporal independence of the captured pictures, a threshold of 30 min was used as the interval to separate two different observations [42]. The data processing was done within the Feline Research Center of National Forestry and Grassland Administration of the Northeast Forestry University by experts in Chinese carnivores who made visualization to identify species. All animal presence was recorded, and we only used specific photographs (for three carnivore species) in this study.

#### 2.2.2. Predictor Variables

To formulate a fit candidate model set, predictor variables were selected according to the observed landscape features. Three variables in two categories were collected. Elevation (el) was an environmental variable. Distance from villages (dv) and distance from roads (dr) were spatial measures of anthropogenic disturbances. The distance measurements were made by the proximity-near tool from the geoprocessing analysis menu in ArcMap (ArcGIS version. 10.2.0.3348, copyright^©^ 1999–2013 Esri Inc. Redlands, USA, all rights reserved) after the projection of all villages and digitized roads inside and outside the research area. Software performance was improved by standardizing the continuous values of the variables with a z-transformation [38] as follows:

(1) $$x_{i}=\frac{x_{1}-a}{b}$$

where $x_{i}$ is the new covariate value, $x_{1}$ is the original observed covariate value, a is the average of all the covariate values, and b is the standard deviation.

To test for multicollinearity among predictor variables, Pearson’s correlation coefficient (r) was calculated in R v. 3.5.0 [43,44]. Covariates were strongly linked when r > 0.6. The total numbers of survey days were not equal for all seasons. Hence, data for three seasons were aggregated in the same length periods in order to standardize the number of surveys and improve software model computation performance [16,45]. For Seasons 1 and 3, two weeks were considered as one survey, and for Season 2, ten days were aggregated. A species was considered to be detected if it was present at a site in an interval of aggregated days. Otherwise, it was not detected [16,45].

### 2.3. Data Analysis

#### 2.3.1. Occupancy Models

Occupancy models were run in PRESENCE v. 5.8 (<130315.0823>; James E. Hines, Dunedin, New Zealand), to investigate multi-year species dynamics. Occupancy model construction varies with the type of research hypothesis [10]. However, all models use presence/absence data as the input and obtain specified estimates as the output. Occurrence data for targeted species were collected at different sites within an area. As species detection was imperfect, data were collected within N sites for T sampling occasion and species presence/absence data were recorded while checking cameras [22,38]. Occupancy models can make strong inferences about the effects of landscape fluctuations on species site occupation [10]. These models are likelihood-based, and their estimates may be modeled as covariate functions [46]. Thus, each sampling site was associated with the averages of various predictor variables and their influences were modeled on the estimated species parameters.

#### 2.3.2. Dynamic Multi-Season, Single-Species Model

**Figure 2:**
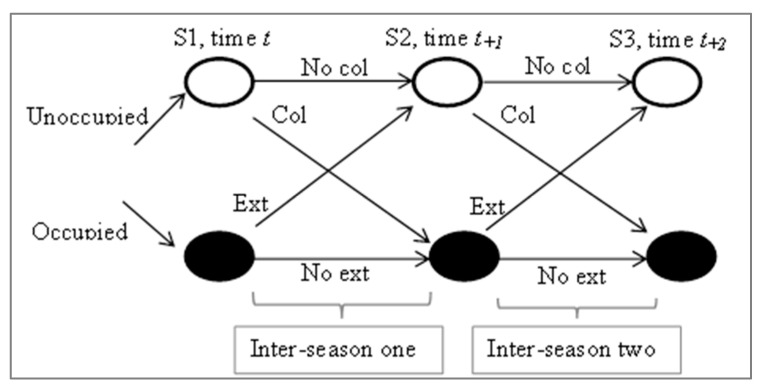
Scheme of multi-season process models, Col, colonization; Ext, extirpation; No col, no colonization; No ext, no extirpation.

In the present study, season refers to the year wherein consecutive surveys were conducted. A logistic model was computed according to the method of MacKenzie et al. [22]. Four parameters that may change between years were used: (1) occupancy (psi) (probability of a site being occupied by a species); (2) detection (p) (probability of a species being detected at a site; (3) colonization (gam) (probability of a site being unoccupied during time t and becoming occupied at time t_+1_); and (4) extirpation (eps) (probability of a site previously occupied during time t becoming unoccupied at time t_+1_) [22,46]. These parameters encompass all dynamic patterns within a habitat over the predetermined time t_+n_ (Figure 2), where n = different seasons.

A derived parameter, the rate of change in occupancy, was performed to interpret the species dynamics. This estimate was computed according to MacKenzie et al. [34]:

(2) $$\lambda \prime_{t}=\frac{\psi_{t+1}/\left(1-\psi_{t+1}\right)}{\psi_{t}/\left(1-\psi_{t}\right)}$$

These dynamic multi-seasons, single-species models use a strong design. Estimates (psi, p, gam, and eps) are assumed to be “closed” to changes or movement during the surveys. In practice, however, they may be “open” between seasons [34]. This assumption introduces heterogeneity, which, in turn, causes estimate bias. Nevertheless, predictor variables can overcome this bias [22]. Therefore, models were constructed using different predictor variables to determine whether estimates across seasons were influenced by year, elevation, distance from villages, or distance from roads. An information-theoretical approach was developed to model estimates over three years. The set of potential models was reduced with Akaike’s Information Criterion (AIC) [47]. Parameters that best described the detectability of each species per year were selected. The parameters “specific site effects” and “seasonal effects” were represented in the models by “sse” and “se”, respectively; they were used to perform detectability. Thus, predictor variables were used to compute psi, gam, and eps, and the considered estimates were from model averages made from all the models computed. This multi-stage procedure was realistic because the primary objective was not to estimate detectability. However, detection was important for the determination of occupancy estimation values [10,48]. Models with ∆AIC ≤ 6.0 were suitable for the prediction of relevant estimate inferences. The 2.0 threshold was too stringent [49], and a 6.0 cutoff was considered apt [50]. Trends in species occupancy progression were predicted according to various seasonal values. The “occupancy equilibrium” trend for each species was calculated following MacKenzie et al. [34]:

(3) $$\psi_{equilibrium}=\frac{\gamma }{\left(\gamma +\varepsilon \right)}$$

To illustrate the influences of predictor variables on vital species estimates such as colonization and extirpation, maps of the strong variable effects were plotted in ArcGIS v. 10.2.

#### 2.3.3. Dynamic Multi-Season Co-Occurrence Model

The co-occurrence of three carnivores was investigated over three years and multi-season, two-species occupancy models were run. NCL was designated Species A (dominant) and LC and RF were designated Species B (subordinate). For the second combination, RF was Species A and LC was Species B. We computed seven co-occurrence estimates (Table 1). The φ output illustrated species interactions within a habitat: φ < 1 indicated species avoidance, φ > 1 indicated species overlap, and φ = 1 indicated independent species co-occurrence. The occupancy probability and vital probabilities (colonization and extirpation) in the co-occurrence model across years were selected [31,41]. Values were estimated to determine how the three aforementioned species shared this habitat. We made a co-occurrence model with species two-by-two instead of three species together because there are still issues to perform a multi-season–multispecies model (MacKenzie, pers. comm.) 

## 3. Results

### 3.1. Naïve Occupancy, Trap Success, and Seasonal Capture

Surveys in the TNR generated 589, 496, and 472 independent photographs for targeted species in Seasons 1–3, respectively. There were 10,530, 7378, and 8308 night-traps for the three respective sampling periods. Species photographs were apportioned according to the number of cameras and the duration of data collection per season (Table 2). However, Sampling Period 1 used more cameras than those for Sampling Periods 2 and 3 but there were fewer photographs of LC than in the last two sampling periods.

### 3.2. Multi-Year Estimates and Variable Effects

We fitted eight models for RF and nine models each for LC and NCL. All models strongly upheld our inferences. Specific site effects and seasonal effects explained variations in detection probability because models with p(sse) [38] and p(se) in detection computation had lower ∆AIC than those with both variables. All models had ∆AIC that were inferior to the cutoff we selected (Table 3). For RF, the model with the lowest ∆AIC was psi(.)gam(year),eps(year),p(sse). For LC, it was psi(.),gam(.),eps(year+dr),p(se). For NCL, it was psi(year+dr),gam(year+dr),p(se).

**Figure 3:**
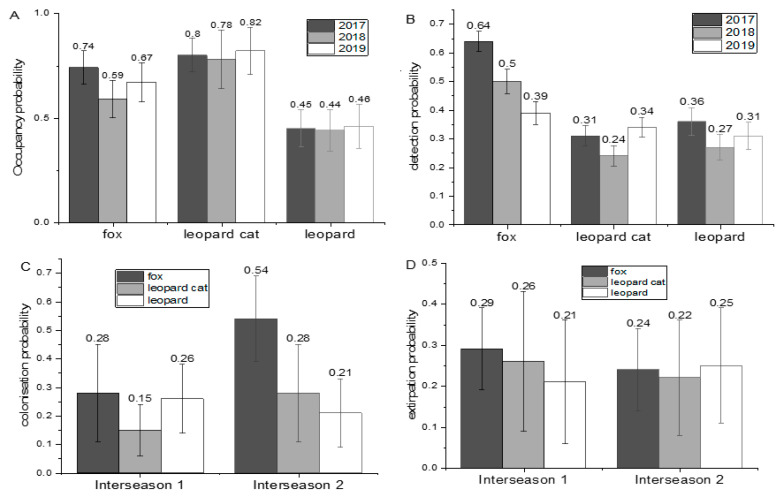
General independent estimated trends of simpler models (with only year variations) for RF, LC, and NCL across the TNR from 2017 to 2019: (**A**) occupancy probability; (**B**) detection probability; (**C**) local colonization in two interseasons; and (**D**) extirpation in two interseasons within study area (numbers above bars are estimate’s values).

For models with annual variations and across all seasons, LC had the highest general probability of occupancy (especially in Sampling Period 3 (0.82 ± 0.11)) followed by RF and NCL (Figure 3A) with an average occupancy for NCL of 0.44 ± 0.10 in Season 2. RF was most frequently detected throughout all sampling periods (Figure 3B). Its peak was 0.64 ± 0.03 in 2017. The detection of NCL had an acceptable value in all sampling periods. Its maximum detectability was 0.36 ± 0.03 in 2017. LC was the least often detected and its lowest detection (0.24 ± 0.03) occurred in Season 2. RF had the highest local colonization (Figure 3C) during Interseason 2 (0.54 ± 0.15) followed by LC in the same interseason (0.28 ± 0.17). NCL had the second highest colonization probability for Interseason 1 (0.26 ± 0.12). The extirpation (Figure 3D) were nearly constant for all species. RF and LC presented with relatively less variation in population decline between the interseasons (0.29 ± 0.10 to 0.24 ± 0.10 and 0.26 ± 0.17 to 0.22 ± 0.14, respectively). For NCL, there was a small increase in extirpation from 0.21 ± 0.15 to 0.25 ± 0.14.

**Figure 4:**
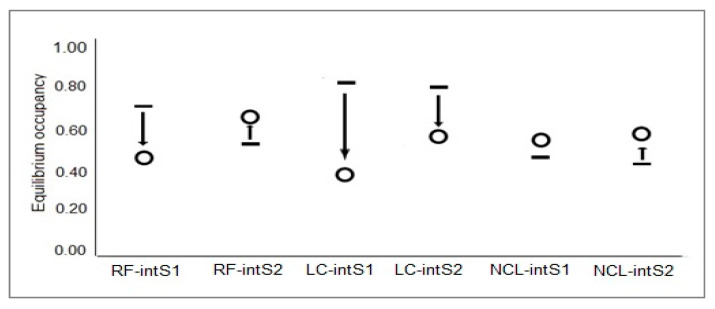
Occupancy equilibrium (O) in relation to occupancy (▬) of year before interseason over three years within the TNR. Arrows indicate degree and trend of change in occupancy. RF-intS1, red fox Interseason 1; LC-intS1, leopard cat Interseason 1; NCL-intS1, North Chinese leopard Interseason 1; RF-intS2, red fox Interseason 2; LC-intS2, leopard cat Interseason 2; NCL-intS2, North Chinese leopard Interseason 2.

All three species displayed average rates of change in occupancy (0.50, 0.88, and 0.96 for RF, LC, and NCL in Interseason 1 and 1.41, 1.28, and 1.08 for RF, LC, and NCL in Interseason 2). NCL had good occupancy equilibrium and a slight increase during Interseason 2. RF had an average equilibrium which consecutively decrease and increase in interseasons. LC demonstrated a decrease in occupancy equilibrium over all two interseasons (Figure 4).

All other predictor variables (elevation and distance from villages and roads) inserted into the models influenced the estimates. Detectability was computed from seasonal and seasonal site effects. The influences of the variables were identical across all three sampling periods (Table 4). 

For all three years, all species presented with diverse estimate values per sampling period and site (Table 5; maxima and minima). Elevation was positively correlated with RF and LC occupancy but negatively correlated with NCL occupancy during all three sampling periods. Distance from villages positively influenced occupancy except for LC. Distance from roads was positively correlated with the occupancy of all three species. Except for NCL colonization, the extirpation and colonization probabilities were positively influenced by distances from villages and roads.

**Figure 5:**
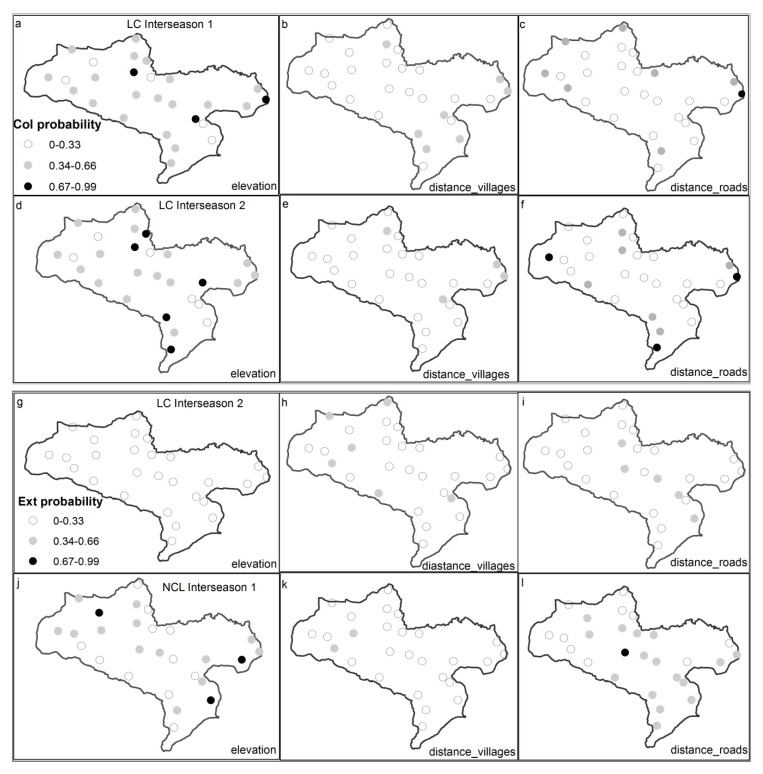

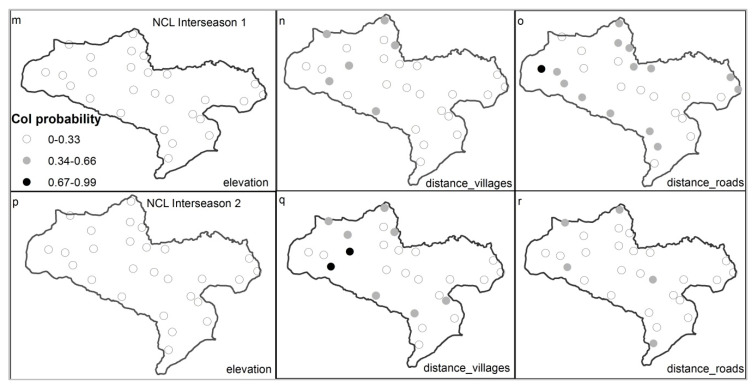
Differences between effects of elevation and distance from villages and roads on local colonization and extirpation of LC and NCL in the TNR, (**a**–**i**) are maps of the leopard cat and (**j**–**r**) are maps for the North Chinese leopard. Proportions in legend are applicable to all maps. The same line represents variables in the same interseason for species on the left map in the line. Col and Ext probability mean, respectively, colonization and extirpation probability (each dot on these maps represents one sample site and only significant differences were mapped).

The environmental factor and anthropogenic disturbances slightly influenced all estimates for RF (occupancy and colonization positively while the extirpation was negatively impacted). For LC, only occupancy was weakly impacted. The effect of elevation was stronger than those of the anthropogenic disturbances (villages and roads) on LC colonization in both interseasons (Figure 5a–f). For the extirpation of LC during the Interseason 2, there was no distinct difference of the impact of the interaction between environmental and anthropogenic disturbances effect, but with a slight evidence for anthropogenic disturbances (Figure 5g–i). For the NCL, occupancy was slightly impacted by variables used. The extirpation was affected by both elevation and distance from roads more than it was by distance from villages during the Interseason 1 (Figure 5j–l). Distances from roads markedly affected its colonization compared to the effect of elevation in the Interseason 1 (Figure 5m–o). In the Interseason 2, distance from villages had a greater influence than elevation and distance from roads (Figure 5p–r).

### 3.3. Multi-Year Assessment of Species Co-Occurrence

LC showed positive co-occurrence with RF and independent cohabitation (φ ~ 1 everywhere; Table 6). It had comparatively higher occupancy at sites where RF was detected during the first two sampling periods. RF colonization and extirpation probabilities were relatively higher in the presence of LC during both interseasons (gamAB and gamAb; Table 6). RF had true co-occurrence (φ > 1) with NCL in all seasons and comparatively higher occupancy at sites where NCL occurred only in season one. NCL colonization was relatively higher in the absence of RF (gamAb; Table 6). LC tended to avoid NCL (φ < 1) during sampling period one and occurred independently of the other two interseason (φ ~ 1). The LC occupancy was high in the absence of NCL in season one (psiBA; Table 6). NCL colonization and extirpation were comparatively higher during Interseason 1 in the absence of LC and during Interseason 2 in the presence of LC. All coexistence model combinations described a crescent-shaped φ over 3 years. The lowest φ was 0.93 ± 0.10 and the highest φ was 1.67 ± 0.18 for NCL-LC and NCL-RF, respectively.

## 4. Discussion

### 4.1. Species Estimates across Years

We built generic models wherein variations were computed according to year and seasonal RF and site-specific LC and NCL effects (Table 3). The model outputs indicated that species occupancy did not markedly fluctuate over time. Species occupancy is useful for predicting species distribution, providing relevant inferences [10,39,51]. The constancy of occupancy of NCL, RF, and LC in the TNR demonstrated the stability of the factors that govern the dynamic process by which species adjust to various landscape perturbations due to human activities. Sustainable management practices (afforestation, compensation for livestock depredation, regular wildlife monitoring, etc.) form the basis for this stability. The protected area of this study has benefited from recent Chinese government policies under the Natural Forests Protection Program for landscape restoration and the improvement of management strategies. These measures have increased the size and enhanced the value of the forests and protected areas [52,53]. In the TNR landscape, the NCL is an umbrella species [3]. Its protection will augment the survival of other carnivores such as RF and LC [5,23].

MacKenzie [38] analyzed occupancy models with 40 sampling stations and proposed that site occupancy estimates are usually unbiased at detectability >0.3. They recommended ≥5 sampling occasions. We disclosed detection probabilities >0.3 during all sampling periods for RF and within Sampling Periods 1 and 3 for LC and NCL. During Sampling Period 2, however, the detection probabilities were 0.28 ± 0.03 and 0.24 ± 0.04 for LC and NCL, respectively. According to the number of sampling sites, the dataset was adequate. Based on the conclusions of Nicholson and Van Manen [10] (one season depicts a detectability <0.30 but with more samplings periods), the estimates would be considered accurate. The detection probability value determines occupancy veracity especially when sampling sites and incidences are small [34,48]. Nevertheless, the species presented with an average rate of change in occupancy. There were big values in Interseason 2 and only RF’s values were smaller than NCL and LC’s values in Interseason 1. The stability of NCL occupancy was also evident.

Low rates of change in occupancy might indicate a positive trend in the site occupation of species within this protected area. However, this trend may not remain unchanged as species- and habitat-level dynamics are continuous in landscapes [2,3]. We only used a three-year dataset. Thus, generalization of species stability estimates could be inappropriate. Hence, the output of the present study may constitute preliminary findings for these species’ dynamic within the TNR. The increase in colonization and decrease in extirpation for RF and LC between both interseasons corroborate the theory of positive dynamics for these species in the TNR. NCL colonization declined from 0.26 ± 0.12 to 0.21 ± 0.12 between interseasons. On the other hand, occupancy during the next sampling period might not necessarily be lower than that in the previous period. The NCL extirpation probability was higher than that of the colonization probability in the Interseason 2. However, the occupancy nonetheless increased in Season 3 because these dynamic processes (extirpation and colonization) affect exclusively occupied and unoccupied entities, respectively [38]. NCL occupancy in Season 2 was estimated to be 0.42 ± 0.10. Because the NCL occupancy was <0.50, there will be more unoccupied entities for colonization than the total number of occupied entities where the species would be locally extinct.

### 4.2. Predictor Variable Effects

Heterogeneity within sampling sites and estimation bias may result from species movement among various sites within a sampling period [10,22,34]. We included the anthropogenic variables to look at the impact human activity has on the occupancy of these species. The output of our fit models indicated that the effects of the variables remained unchanged across all seasons for all three species. Accordingly, the effects of the variable “year” and the environmental factor or anthropogenic disturbances are additive and could be notated as “year+el”, “year+dv”, and “year+dr” for all species [38]. The magnitudes of the variable effects might differ between interseasons. Differences were apparent only for colonization and extirpation. The fit models revealed that sites at high elevation had higher RF and LC occupancy and colonization probabilities. This probability of RF and LC occupancy at elevated sampling sites did not preclude their presence at lower elevations. Distance to villages and roads had a greater influence on occupancy compared to elevation. The TNR has homogeneous environmental conditions in its big parts, does not present alternate possibilities of habitat features to wildlife. Species within such areas may also require more auspicious habitat conditions. Otherwise, they might settle in any part of the landscape and have high occupancies there regardless of the effects of environmental factors [12]. The ubiquity of RF [26] and LC [29] might be relevant to their occupancy probabilities. Here, only the small-bodied carnivores RF and LC were associated with the high-altitude sites. Large felids such as jaguar (*Panthera onca*) and cougar (*Puma concolor*) may also occupy areas at high elevation. In fact, these animals have been detected at > 1800 m.a.s.l. for a study in South America [54] which contradict our findings about the NCL. Habitat structure and vegetation type strongly determine occupancy of carnivore species [12,55].

NCL preferred sites remote from human settlements (villages and roads). Carnivores, especially large ones prefer high densities of vegetation cover [54], prey availability [14,23], and quiet, hidden places [55]. Here, NCL colonization slightly increased at sites near villages. These areas are always in contact with carnivores. Nevertheless, low human tolerance for the presence of felids might alter the occupancy of these species in areas surrounding human settlements and threaten carnivore population dynamics [31]. Colonization is a conditional occupancy [38]. Therefore, species adjust their behavior in order to gain access to these sites instead of occupying the vicinity of villages [56,57]. Entry into these areas might enable the animals to prey upon livestock herds. Seasonal livestock proliferation within a part of the landscape during a sampling period might account for the renewed presence of a large carnivore and could be perceived as colonization. These patterns are common to numerous protected areas hosting both carnivores and livestock herders [16,55]. The villages seem to become a sink for the NCL population, and carnivores are alternately attracted in their vicinity. The attraction is based on the proliferation of easy catching prey which enhances human–carnivore interactions. Across years such situation would have serious consequences on species population of NCL in this region since the transformation of a proportion of leopard to sink population status will be permanent if nothing is undertaken by reserve’s managers. However, RF reacted identically as NCL to human settlements. This behavior may result in spatial overlap with NCL in sites remote from villages. The LC is closer to villages in the TNR, and its occupancy increased as distance from villages increased. Therefore, the LC interacts with villagers’ activities and can prey on poultry or small domestic mammals as the species selects areas with prey that are easy to catch rather than those with high population density [27]. An investigation carried out on LC–human interactions could validate this assumption for TNR. This type of contact influences even small- or medium-size species distributions within a landscape [16,58]. There are few published studies of anthropogenic pressure on small carnivores and the effects and mechanisms involved are poorly understood [31,59,60,61].

In contrast, variable effects on the local species extirpation probabilities were lower than those for colonization. Hence, the variables had different effects. Human presence remains a potential threat to consistent and efficacious management in protected areas [6,8,16]. Both environmental and anthropogenic factors shape landscape features [12]. Anthropogenic disturbances may markedly affect species distribution via habitat degradation [4,5], prey depletion [3,14], direct attack on carnivore species [4,14,62], and pathogen transmission [63,64] mainly via invasive domestic carnivores.

### 4.3. Multi-Year Species Coexistence

The general trend of independent co-occurrence over time for RF and LC (φ ~ 1; φ = 1.20 by Season 3; Table 6) may be the result of classic niche dissociation. Both species can be categorized as mesocarnivores but ecological similarity is the most important factor when referring to the niche theory [3,65]. Two species ecologically similar could not continuously coexist because of competitive exclusion [66]. On the other hand, the situation is often highly complex, and competition might persist during coexistence. Competitor species avoid overlapping activity by shifting their environmental space [67,68]. Despite ecological similarity, various factors facilitate independent coexistence. These include human disturbances [30], seasonal resource availability, and changes in seasonal features [69]. In RF-LC co-occurrence, LC was subordinate in the presence of RF when the colonization and extirpation probabilities of the latter were relatively higher during both interseasons where LC was present.

RF predominated in the RF-LC combination but was subordinate to NCL (φ > 1). Only this combination corroborated the hypothesis of total co-occurrence. The TNR landscape is conducive to the coexistence of these species [23]. In most protected areas with anthropogenic activity, coexisting carnivore populations may occupy various habitats by alternating between human settlements and core protection areas. When a community combines dominant and subordinate species, the former may exclude the latter from other sites [3,70]. Although RF was subordinate, NCL had the highest extirpation (epsAB: 0.28 ± 0.11) during the interseason wherein it concomitantly appeared with RF (Table 6). In Season 1, its co-occurrence occupancy (psiBA: 0.55 ± 0.11) was comparatively higher in the presence of NCL. When the activities of dominant and subordinate species overlap, the latter may decrease the fitness of the former [32,33] and raise its extirpation probability [34,35]. LC avoided NCL during season one both co-occurred independently during the last two seasons. Subordinate species may practice avoidance behavior to refrain from entering sites with high prey density preferred by dominant species [71,72]. Here, this avoidance response did not indicate a total lack of contact. LC occupancy was high and acceptable (0.77 ± 15) in the presence of NCL [34]. Species co-occurring less frequently than expected imply the resource partitioning [39] mainly at the food habit rather than the spatial level, since in the latter case, these species somewhat co-occurred [41]. Nonetheless, small-scale dissimilarities in landscape use are still possible.

## 5. Conclusions

Estimating species dynamics with occupancy models is an ancillary approach to species density and abundance studies. It determines the status of species and tracks variations in their distributions via camera traps [10,39]. Here, we performed a multi-year assessment of the coexistence mechanisms for three carnivore species within the TNR. The environmental factor used affected positively mesocarnivores’ occupancy when it decreased the leopard’s estimate. Anthropogenic disturbances were focal predictor variables for LC and NCL colonization and extirpation over time. In our study area, managers should maintain the forest landscape and restore degraded areas. Restored habitats will increase the probability of spatial partitioning and reduce interactions because the φ values between carnivores studied increased with time. Such studies portray species distribution in a landscape and the long-term success of wildlife management depends upon accurate population size data and dynamic predictions. These findings might constitute additional factors to be considered in the plans for carnivore conservation in the TNR. Imbalances in species coexistence threaten the equilibria of endangered and endemic species with average occupancy such as NCL. When they are implemented, occupancy studies should account for various local disturbance sources, plan for the protection of regional species, and enable them to withstand disturbance pressures. Supplementary longer-term studies including species food habits are encouraged at the same sites in order to predict future trends more accurately.

## Figures and Tables

**Table 1:** Parameters interpreted from dynamic multi-season models illustrating dynamic and co-occurrence estimates for RF, LC, and NCL in the TNR.

Parameters	Definitions
psiBA	The probability that Species B initially occupies the area, given that Species A is also present
psiBa	The probability that Species B initially occupies the area, given that Species A is not present
gamAB	The probability that Species A colonizes the area in the interseason t-(t_+1_) given that Species B is present in season t
gamAb	The probability that Species A colonizes the area in the interseason t-(t_+1_) given that Species B is not present in season t
epsAB	The probability that the area goes extinct by Species A in the interseason t-(t_+1_) given that Species B is present in season t
epsAb	The probability that the area goes extinct by Species A in the interseason t-(t_+1_) given that Species B is not present in season t
φ (phi)	Species Interactions Factor (SIF)

**Table 2:** Independently captured photographs of RF, LC, and NCL in the TNR during three sampling seasons, including sampling period (SP); number of cameras used (nCAM); total independent photographs captured (TIP); number of independent photographs (nIP); survey duration representing total number of camera traps on field (SD); global trap success (GTS), for three species = total number of photographs/total number of night traps, and the total number of night traps = number of cameras × days of data collection; specific trap success (STS); and naïve occupancy (NO) performed by number of sites in which species occurred/total number of sites wherein the survey was conducted. Values in bold font are highest in category.

SP	nCAM	TIP	SD (days)	GTS (%)	Fox			Leopard Cat			Leopard		
					nIP	STS	NO	nIP	STS	NO	nIP	STS	NO
One	**81**	**589**	130	5.60	**410**	3.90	**0.74**	108	1.02	**0.77**	**71**	0.67	**0.44**
Two	62	496	119	**6.72**	308	**4.17**	0.60	135	**1.82**	0.55	53	0.71	0.37
Three	62	472	**134**	5.68	259	3.11	0.66	**148**	1.78	0.70	65	**0.78**	0.40

**Table 3:** Top-ranking candidate models for multi-season occupancy analysis for RF, LC, and NCL including AIC values (AICs), delta AIC (∆AIC), AIC weight (W), model likelihood (ML), number of parameters (K), and −2LogLike (−2L).

Models	AICs	∆AIC	W	ML	K	−2L
A. red fox						
psi(.)gam(year),eps(year),p(sse)	743.69	0.00	0.3350	1.0000	32	769.69
psi(.),gam(.),eps(year+dr),p(sse)	744.73	1.04	0.1991	0.5945	32	680.73
psi(year),eps(year+dv),p(sse)	745.67	1.98	0.1245	0.3716	33	679.67
psi(year+el),gam(year+el),p(sse)	746.21	2.52	0.0950	0.2837	34	678.21
psi(.),gam(year+el),eps(year+el),p(sse)	746.28	2.59	0.0917	0.2739	34	678.28
psi(year+dr),gam(year+dr),p(sse)	747.18	3.49	0.0585	0.1746	34	679.18
psi(year+dv),gam(year+dv),p(sse)	747.48	3.79	0.0503	0.1503	34	679.67
psi(.),gam(year+dv),eps(year+dv),p(sse)	747.67	3.98	0.0458	0.1367	34	679.67
B. leopard cat						
psi(.),gam(.),eps(year+dr),p(se)	640.97	0.00	0.4943	1.0000	8	624.96
psi(year),gam(year),p(se)	642.72	1.75	0.3280	0.5945	8	628.72
psi(.),gam(.),eps(year+dv),p(se)	643.76	1.98	0.1950	0.4169	8	627.76
psi(.),gam(year+dv),eps(year+dv),p(se)	645.19	2.47	0.0954	0.2908	10	625.19
psi(year),eps(year),p(se)	645.20	2.48	0.0949	0.2894	8	629.20
psi(year+el),gam(year+el),p(se)	645.81	3.09	0.0700	0.2133	10	625.81
psi(.),gam(year+el),eps(year+el),p(se)	646.73	4.01	0.0442	0.1347	10	626.73
psi(year+dv),gam(year+dv),p(se)	647.28	4.56	0.0336	0.1023	10	627.28
psi(year+dr),gam(year+dr),p(se)	648.69	5.97	0.0166	0.0505	10	628.69
C. North Chinese leopard						
psi(year+dr),gam(year+dr),p(se)	450.35	0.00	0.2726	1.0000	10	430.35
psi(.),gam(year+el),eps(year+el),p(se)	450.32	0.17	0.2504	0.9185	10	430.52
psi(.),gam(year),eps(year),p(se)	452.21	1.86	0.1076	0.3946	8	436.21
psi(year),gam(.),p(se)	452.37	2.02	0.0993	0.3642	7	438.37
psi(year+dv),gam(year+dv),p(se)	452.62	2.27	0.0876	0.3214	10	434.62
psi(year+el),gam(year+el),p(se)	453.28	2.93	0.0630	0.2311	10	433.28
psi(.),gam(.),eps(year+dr),p(se)	453.51	3.16	0.0561	0.2060	8	437.51
psi(year),eps(year+dv),p(se)	454.98	4.63	0.0269	0.0988	9	436.98
psi(.)gam(year+dv),eps(year+dv),p(se)	455.31	4.96	0.0228	0.0837	10	435.31

**Table 4:** Effects of predictor variables on estimates (occupancy, colonization, and extirpation) of RF, LC, and NCL in the TNR used in candidate set models; ++ represents a positive correlation and + − a negative correlation. Influence of variable was identical across all three sampling periods.

Season: All Years	Occupancy			Colonization			Extirpation		
Variables	RF	LC	NCL	RF	LC	NCL	RF	LC	NCL
Elevation	++	++	+ −	++	++	+ −	+ −	+ −	++
Distance from village	++	+ −	++	++	+ −	+ −	+ −	++	++
Distance from road	++	++	++	++	++	++	+ −	+ −	+ −

**Table 5:** Estimates of occupancy (psi), local colonization (gam), and extirpation (eps) for RF, LC, and NCL in the TNR derived from multi-season, single-species models with predictor variable effects. Only maxima and minima are presented (el, elevation; dr, distance from roads; dv, distance from villages).

			Occupancy (psi)			Colonization (gam)			Extirpation (eps)		
			el	dv	dr	el	dv	dr	el	dv	dr
RF	2017	max	0.84 ± 0.09	0.78 ± 0.15	0.85 ± 0.12	0.53 ± 0.21	0.30 ± 0.20	0.57 ± 0.23	0.42 ± 0.12	0.31 ± 0.17	0.33 ± 0.15
		min	0.58 ± 0.18	0.71 ± 0.08	0.64 ± 0.07	0.12 ± 0.14	0.20 ± 0.13	0.21 ± 0.14	0.20 ± 0.23	0.26 ± 0.10	0.22 ± 0.13
	2018	max	0.74 ± 0.08	0.61 ± 0.10	0.75 ± 0.09	0.77 ± 0.10	0.58 ± 0.10	0.72 ± 0.10	0.35 ± 0.16	0.25 ± 0.26	0.29 ± 0.10
		min	0.42 ± 0.08	0.55 ± 0.09	0.47 ± 0.09	0.32 ± 0.09	0.50 ± 0.21	0.39 ± 0.31	0.16 ± 0.09	0.23 ± 0.12	0.17 ± 0.21
	2019	max	0.80 ± 0.10	0.71 ± 0.13	0.77 ± 0.08	NA	NA	NA	NA	NA	NA
		min	0.50 ± 0.09	0.63 ± 0.14	0.57 ± 0.14	NA	NA	NA	NA	NA	NA
LC	2017	max	0.80 ± 0.03	0. 85 ± 0.07	0.82 ± 0.05	0.60 ± 0.23	0.44 ± 0.12	0.58 ± 0.13	0.27 ± 0.08	0.54 ± 0.12	0.65 ± 0.15
		min	0.49 ±0.05	0.67 ± 0.03	0.70 ± 0.02	0.32 ± 0.31	0.31 ± 0.23	0.29 ± 0.11	0.25 ± 0.05	0.16 ± 0.06	0.10 ± 0.21
	2018	max	0.85 ± 0.11	0.84 ± 0.15	0.82 ± 0.10	0.69 ± 0.19	0.62 ± 0.10	0.68 ± 0.21	0.22 ± 0.12	0.45 ± 0.10	0.59 ± 0.19
		min	0.54 ± 0.07	0.67 ± 0.18	0.77 ± 0.23	0.41 ± 0.24	0.38 ± 0.13	0.26 ± 0.24	0.21 ± 0.16	0.14 ± 0.08	0.08 ± 0.12
	2019	max	0.85 ± 0.06	0.86 ± 0.1 4	0.83 ± 0.11	NA	NA	NA	NA	NA	NA
		min	0.57 ± 0.11	0.65 ± 0.09	0.70 ± 0.09	NA	NA	NA	NA	NA	NA
NCL	2017	max	0.66 ± 0.01	0.75 ± 0.10	0.75 ± 0.06	0.28 ± 0.21	0.59 ± 0.20	0.34 ± 0.08	0.56 ± 0.04	0.21 ± 0.13	0.48 ± 0.21
		min	0.21 ± 0.10	0.32 ± 0.0 7	0.19 ± 0.09	0.26 ± 0.16	0.13 ± 0.09	0.21 ± 0.08	0.19 ± 0.05	0.10 ± 0.18	0.09 ± 0.16
	2018	max	0.69 ± 0.08	0.78 ± 0.13	0.77 ± 0.05	0.21 ± 0.14	0.73 ± 0.08	0.25 ± 0.09	0.32 ± 0.09	0.35 ± 0.14	0.38 ± 0.20
		min	0.28 ± 0.08	0.30 ± 0.18	0.26 ± 0.08	0.19 ± 0.09	0.12 ± 0.03	0.17 ± 0.12	0.10 ± 0.05	0.12 ± 0.11	0.06 ± 0.26
	2019	max	0.68 ± 0.14	0.79 ± 0.06	0.74 ± 0.18	NA	NA	NA	NA	NA	NA
		min	0.27 ± 0.12	0.30 ± 0.06	0.18 ± 0.12	NA	NA	NA	NA	NA	NA

NA, not applicable (there were two interseasons presented respectively in the table as 2017 and 2018 for colonization and extirpation probabilities).

**Table 6:** Dynamic co-occurrence results for NCL (Species A) and RF and LC (Species B), the probability of occupancy of RF and LC when leopard is present (psiBA) or absent (psiBa), including the leopard colonization and extirpation probabilities when RF and LC are present (gamAB/epsAB) or absent (gamAb/epsAb) during the interseason. The table contains also the species interaction factor (SIF) of species’ pair co-occurrence. Estimates are accompanied by standard errors and NA indicates not applicable because colonization and extirpation probability are present only in interseasons.

Season	psiBA	psiBa	gamAB	gamAb	epsAB	epsAb	*φ*
RF-LC							
one	0.78 (0.08)	0.66(0.27)	0.22 (0.20)	0.20 (0.18)	0.29(0.10)	0.15(0.13)	1.01(0.05)
two	0.81 (0.09)	0.69(0.16)	0.36 (0.12)	0.31 (0.28)	0.22(0.11)	0.19(0.22)	1.11(0.15)
three	0.56 (0.11)	0.63(0.10)	NA	NA	NA	NA	1.20(0.10)
NCL-RF							
one	0.55 (0.11)	0.26 (0.15)	0.38 (0.24)	0.39 (0.24)	0.28(0.11)	0.17(0.18)	1.23(0.12)
two	0.35 (0.12)	0.51(0.11)	0.45 (0.24)	0.47 (0.19)	0.19(0.12)	0.24(0.20)	1.65(0.19)
three	0.62 (0.24)	0.68(0.12)	NA	NA	NA	NA	1.67(0.18)
NCL-LC							
one	0.77 (0.12)	0.82(0.10)	0.42 (0.21)	0.44 (0.47)	0.10(0.20)	0.13(0.20)	0.93(0.10)
two	0.77 (0.15)	0.60(0.20)	0.45 (0.15)	0.39 (0.23)	0.29(0.18)	0.22(0.17)	1.06(0.16)
three	0.63 (0.15)	0.46(0.17)	NA	NA	NA	NA	1.14(0.10)
